# High-fidelity transmission of high-frequency burst stimuli from peripheral nerve to thalamic nuclei in children with dystonia

**DOI:** 10.1038/s41598-021-88114-w

**Published:** 2021-04-19

**Authors:** Estefanía Hernandez-Martin, Enrique Arguelles, Yifei Zheng, Ruta Deshpande, Terence D. Sanger

**Affiliations:** 1 Department of Electrical Engineering and Computer Science, University of California, Irvine, CA USA; 2 Department of Biomedical Engineering, Viterbi School of Engineering, University of Southern California, Los Angeles, CA USA; 3 Department of Neurology, Children’s Health of Orange County (CHOC), Orange, CA USA

**Keywords:** Dystonia, Movement disorders

## Abstract

High-frequency peripheral nerve stimulation has emerged as a noninvasive alternative to thalamic deep brain stimulation for some patients with essential tremor. It is not known whether such techniques might be effective for movement disorders in children, nor is the mechanism and transmission of the peripheral stimuli to central brain structures understood. This study was designed to investigate the fidelity of transmission from peripheral nerves to thalamic nuclei in children with dystonia undergoing deep brain stimulation surgery. The ventralis intermediate (VIM) thalamus nuclei showed a robust evoked response to peripheral high-frequency burst stimulation, with a greatest response magnitude to intra-burst frequencies between 50 and 100 Hz, and reliable but smaller responses up to 170 Hz. The earliest response occurred at 12–15 ms following stimulation onset, suggesting rapid high-fidelity transmission between peripheral nerve and thalamic nuclei. A high-bandwidth, low-latency transmission path from peripheral nerve to VIM thalamus is consistent with the importance of rapid and accurate sensory information for the control of coordination and movement via the cerebello-thalamo-cortical pathway. Our results suggest the possibility of non-invasive modulation of thalamic activity in children with dystonia, and therefore the possibility that a subset of children could have beneficial clinical response without the need for invasive deep brain stimulation.

## Introduction

Cerebral palsy is associated with numerous types of neurological impairment, including dystonia. Some patients with dystonia have benefited from noninvasive methods of nerve stimulation such as transcutaneous electrical nerve stimulation (TENS), which modulates the motor cortex though sensory afferent inputs, and peripheral nerve stimulation (PNS), which acts at the subcortical level^1,2^. However, for children with severe forms of dystonia, deep-brain stimulation (DBS) remains one of the few options for significant improvement^3–6^. While ongoing research on DBS is likely to increase its efficacy and safety, it is nevertheless important to search for noninvasive or minimally invasive methods that could have similar effectiveness.

Dystonia in children is defined as a movement disorder in which involuntary sustained or intermittent muscle contractions cause twisting and repetitive movements, abnormal postures, or both^7^. The mechanism underlying childhood dystonia is unknown, but may include an imbalance between midbrain dopaminergic and striatal cholinergic signaling^8^, abnormal patterns of subcortical activity, excessive basal ganglia or peripheral loop gain, or decreased focusing of intended patterns of muscle activity^5,7,9–11^. Recent work in animals has suggested an important role of cerebellum in some forms of dystonia^12^ and this has been supported by recent reports of benefit from DBS in cerebello-thalamo-cortical pathways in children with secondary dystonia^3,4^. In particular, stimulation in ventralis intermediate (VIM) or ventral posterolateral (VPL) nuclei of the thalamus has been helpful in a subset of children^3^. This raises the possibility that if peripheral noninvasive stimulation could activate VIM, then clinical benefit could perhaps be obtained if appropriate pulse frequencies and patterns can be determined.

Precisely timed motor responses are executed by cerebellar cortex, which responds to the temporal sequences of various sensory inputs with high fidelity^13^. High fidelity includes the ability to detect the rapid onset or offset of proprioceptive or tactile signals. Therefore, one might predict that sensory pathways involved with rapid and accurate motor control would possess relatively high bandwidth. We could potentially leverage such a pathway to provide precise stimulation patterns to thalamic nuclei that receive cerebellar efferents. Few previous studies have investigated the bandwidth and fidelity with which sensory information is transmitted from the periphery to cerebellar thalamic nuclei in humans. This study was designed to investigate the delay and fidelity of transmission from peripheral nerves to thalamic nuclei in order to establish an optimized protocol for testing the use of PNS in the treatment of childhood movement disorders.

## Results

**Figure 1:**
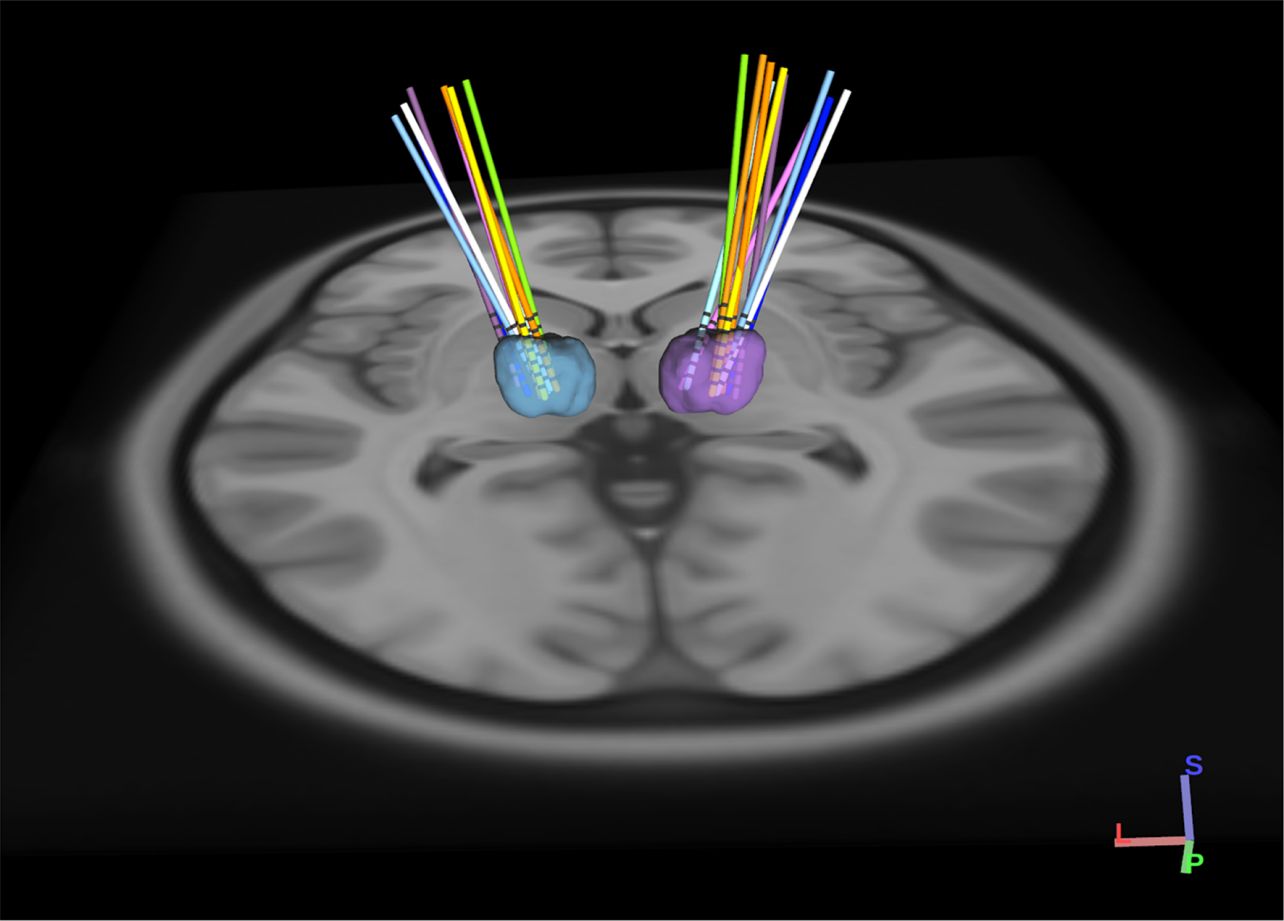
3D rendering of group based DBS electrodes in the bilateral ventral intermediate nuclei. Axial view from normalized scans into MNI space. The VIM boundaries were defined by the DISTAL atlas. Each pair of DBS electrodes corresponds to a patient and are represented by a different color for each patient. A total of 20 DBS electrodes in 10 dystonic patients are represented through DSI Studio, V3 (http://dsi-studio.labsolver.org).

**Figure 2:**
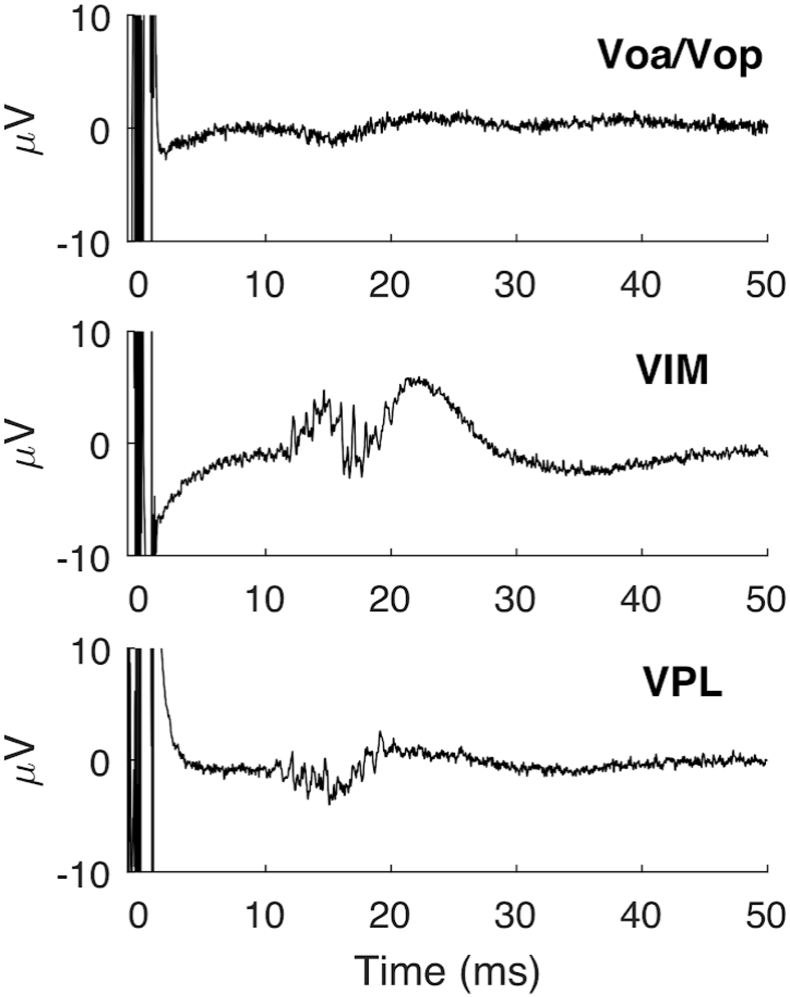
Average thalamic EPs in responses to contralateral single-pulse median nerve stimulation. Note that VIM and VPL EPs have an onset at approximately 11 ms and first peak at 15 ms.

Electrode locations were confirmed by postoperative CT scan, referenced to the preoperative MRI in MNI (Montreal Neurological Institute) coordinates, and rendered in 3 dimensions relative to the location of VIM specified in the DISTAL atlas^14^ (Fig. 1). Primary sensory thalamic nuclei VIM and VPL showed a stronger response to PNS when compared to secondary motor nuclei including the ventral oralis anterior/posterior (Voa/Vop), with mean peak latency in VIM at approximately 15 ms (Fig. 2). As shown previously, the presence of a peripherally evoked response can be used to distinguish sensory from motor thalamic nuclei^15–18^.

Sensory EPs showed two distinctive waveforms, with low- and high-frequency components. These results are consistent with previous studies in adults, in which the origin of the high-frequency component was associated with the synchronized firing of interneurons, whereas the slow waveform was associated with the depolarization of afferent sensory projections^19,20^. Because the focus of this work was transmission fidelity between peripheral nerves and thalamic nuclei, only the low-frequency waveform was further analyzed.

### Individual analysis

**Figure 3:**
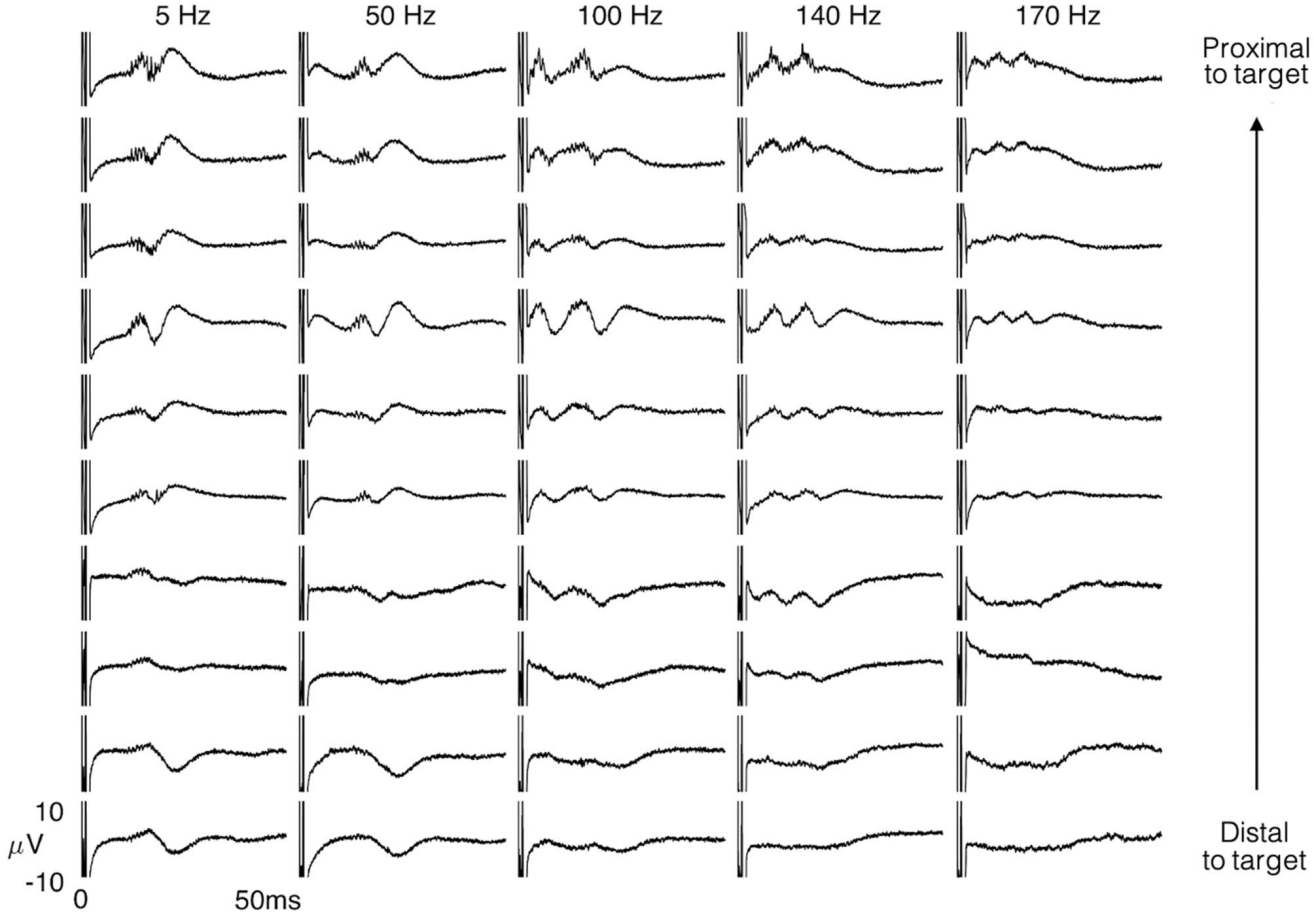
Robust evoked responses are observed in the microelectrodes proximal to the target thalamic nuclei, with a change in the amplitudes as a function of burst frequency. Representative evoked VIM responses are presented for each of the 10 micro-contacts.

Figure 3 shows the evoked response in all contacts in a single VIM electrode in response to burst stimulation at 5 Hz with increasing burst stimulation frequency. Consistent with the location of the recording electrode with respect to the thalamic nucleus, EPs were more robust in the microelectrodes proximal to the target location, compared with those located distally. Robust EPs decreased in amplitude as burst stimulation frequency increased.

**Figure 4:**
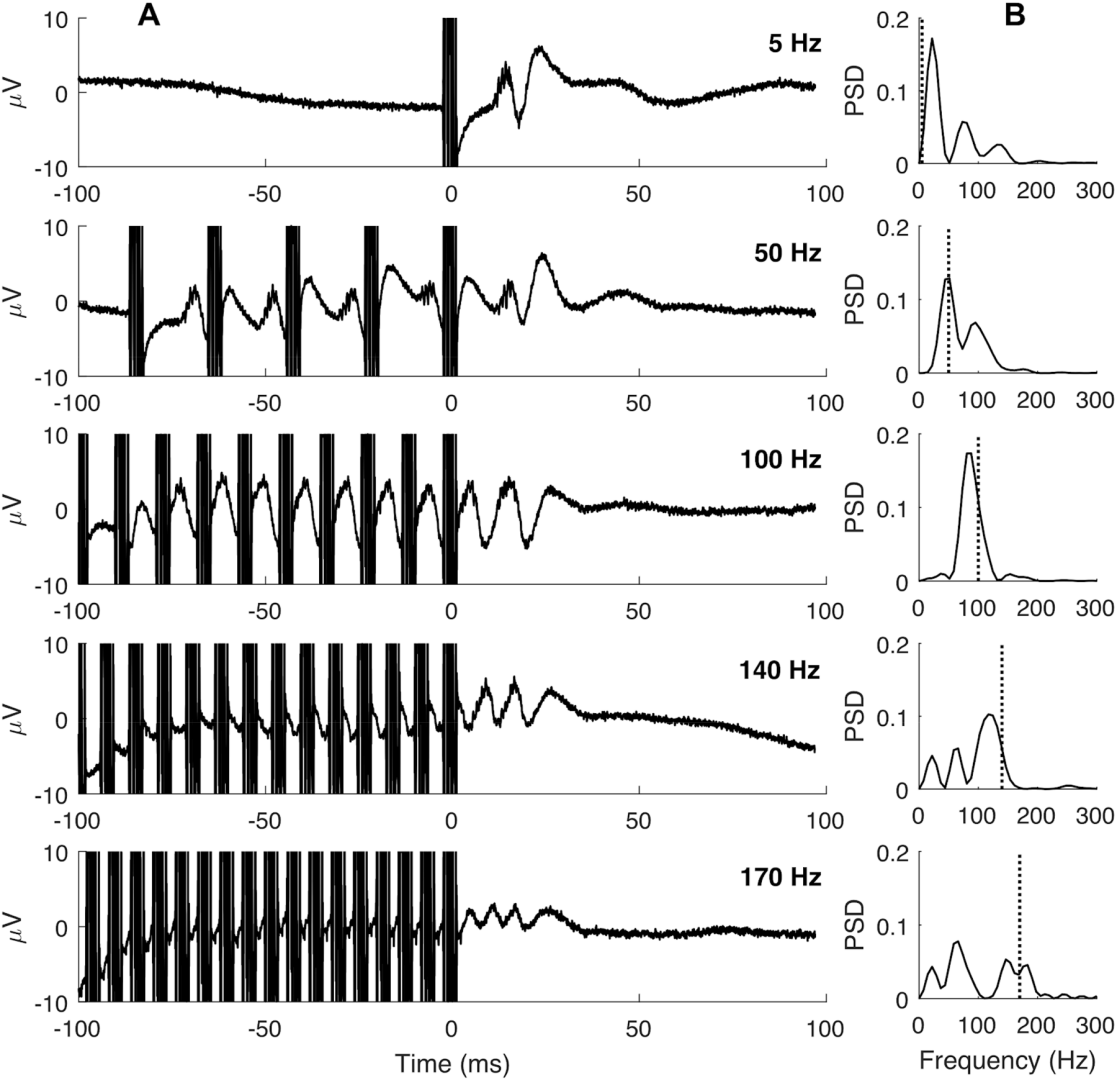
EP in response to a single 5-Hz pulse (top row) and 50–170-Hz burst stimulation for a single VIM contact in a single subject. (**A**) Average traces for the 100-ms stimulation period (negative time values) and the 100-ms EP response used for analysis (positive time values). Stimulation artifacts appear as thick vertical lines. (**B**) Power spectral density (PSD) calculated from the 100-ms period after the last stimulus pulse. Dotted line indicates stimulation frequency.

Only EPs after the final pulse in the burst were considered, because responses during the burst are occluded by the artifact associated with stimulation. Multiple EPs are visible at the highest frequencies due to the second-to-last and third-to-last pulses; evoked responses to each pulse are seen within the burst (Fig. 4A). Figure 4B shows the power spectral density of the EPs for the 100-ms period after the last stimulation pulse, with peaks clustering around the burst stimulation frequency.

**Figure 5:**
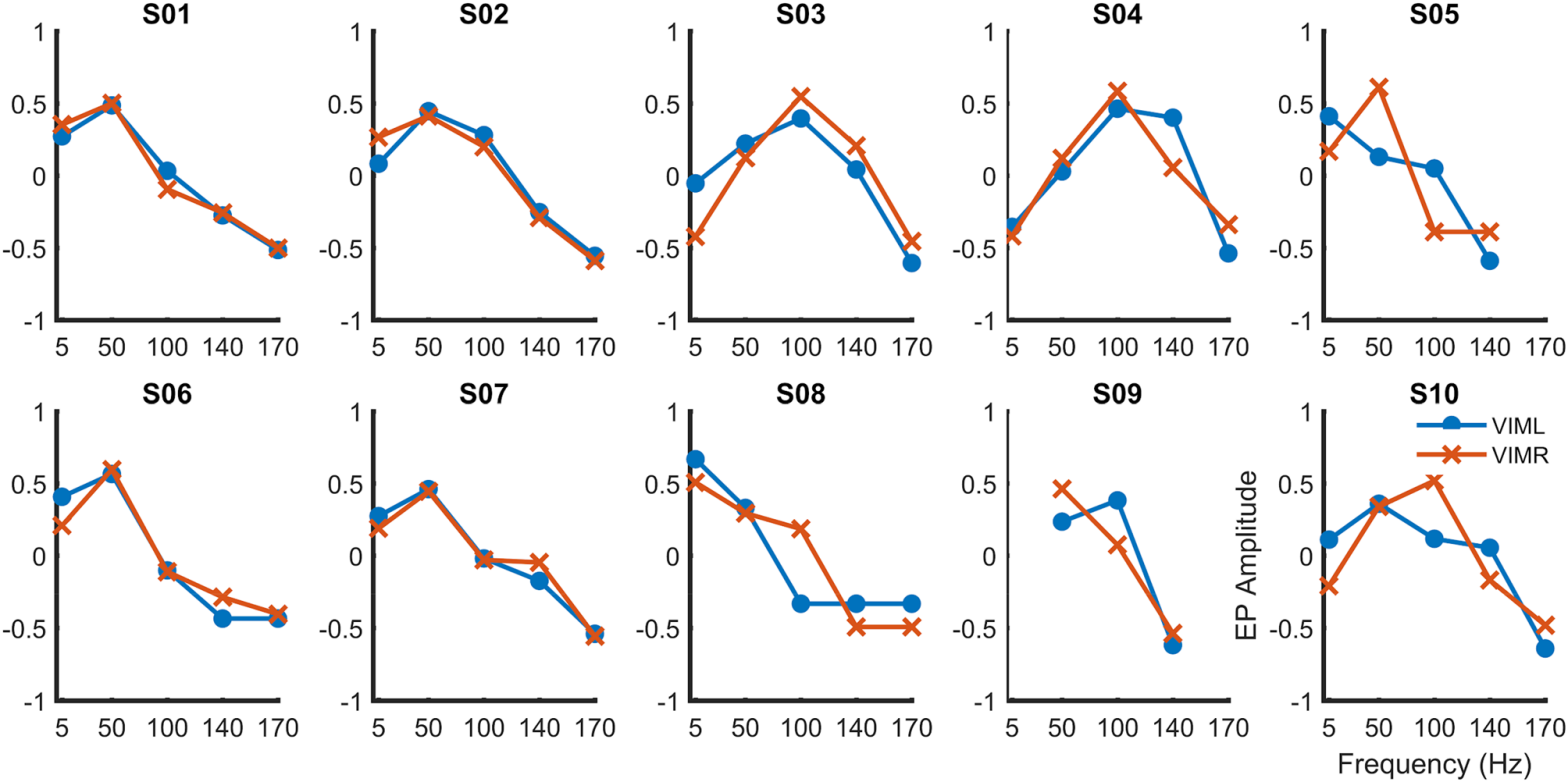
EP amplitudes from left (blue) and right (red) VIM as a function of stimulation frequency during stimulation of the contralateral median nerve, for each subject. Horizontal axes indicate stimulation frequency. Vertical axes represent the normalized EP values.

Figure 5 shows the average magnitude over all 10 subjects. Overall, responses were greatest for pulse frequencies of 50–100 Hz. Peak responses varied among patients, with maxima at 50 Hz, 100 Hz, and, in one case, a single pulse at low frequency (5 Hz). Most of the subjects showed EP responses for all stimulation frequencies, except those subjects who were not able to tolerate high-frequency stimulation.

### Group analysis

**Figure 6:**
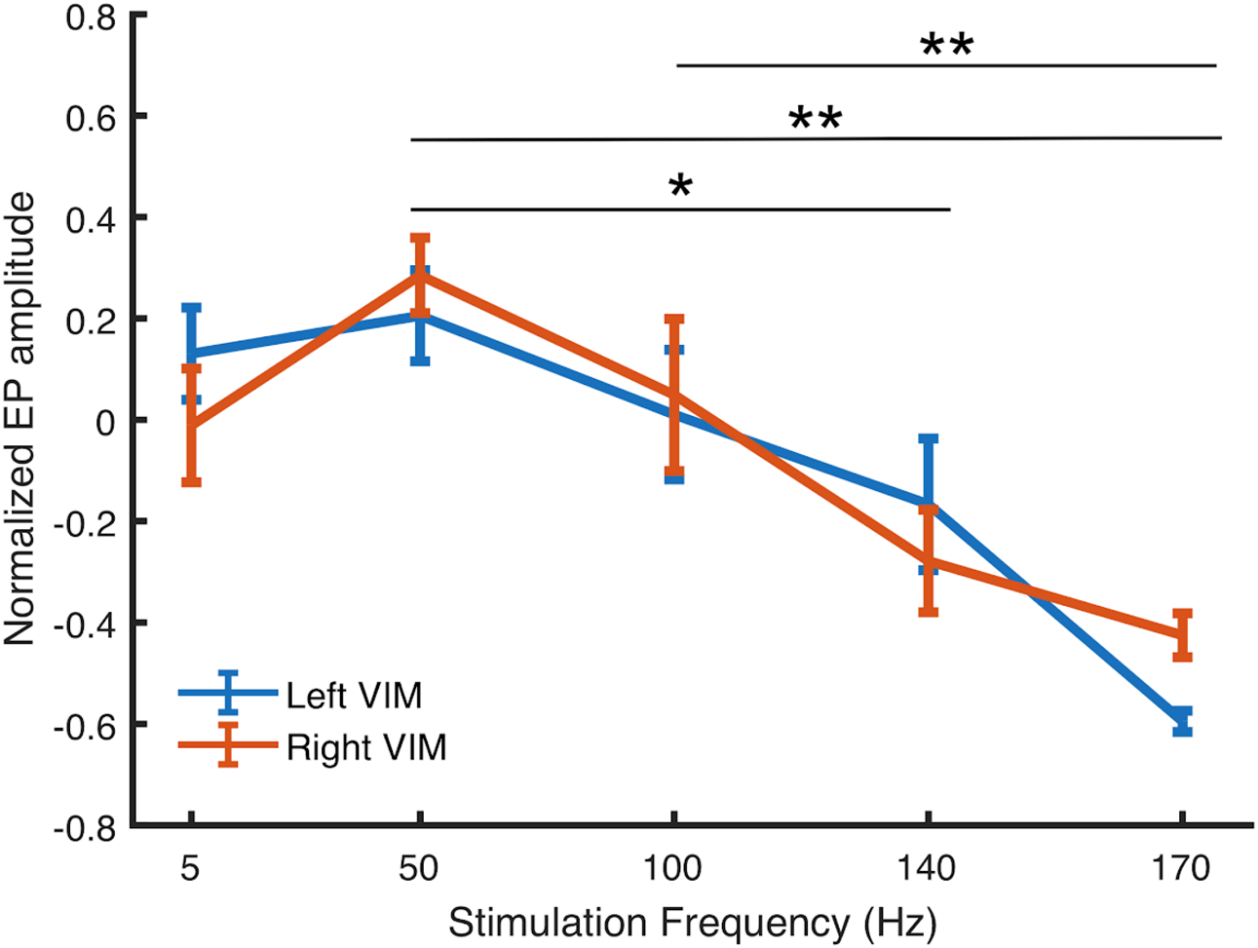
Average EP amplitude as a function of pulse frequency for all subjects. EP amplitude differed significantly between 50 vs. 140 Hz (*p* < 0.01), 50 vs. 170 Hz (*p* < 0.001), and 100 vs. 170 Hz (*p* < 0.001). Error bars indicate standard error.

Figure 6 shows normalized EP amplitude in VIM, averaged over all 10 patients. On average, responses were larger for stimulation frequencies between 50–100 Hz. ANOVA was performed to compare average change in EP amplitude among stimulation frequencies. ANOVA assumptions were tested using Levene's test for homogeneity of variance and the Kolmogorov–Smirnov test for normality. Significant differences were observed between stimulation at 5 vs. 170 Hz; 50 vs. 140 Hz; 50 vs. 170 Hz, and 100 vs. 170 Hz (n = 10, threshold *p* < 0.05, corrected FWER). Mean group EP did not differ significantly at 5 vs. 50 Hz (*p* = 0.22), 5 vs. 100 Hz (*p* = 0.99), 5 vs. 150 Hz (*p* = 0.16), or 140 vs. 170 Hz (*p* = 0.2). There was a significant main effect at 50 Hz, and Dunnett's post-hoc test revealed a significant difference (*p* < 0.001) between the group mean at 50 vs. 140 Hz and 50 vs. 170 Hz. Dunnett's test also revealed a significant difference (*p* < 0.001) between stimulation at 100 vs. 170 Hz.

## Discussion

Our results show frequency-dependent transmission of signal from the median nerve to the VIM nucleus of the thalamus, with accurate representation of frequency up to 170 Hz and peak sensitivity at 50–100 Hz. Transmission delays to the first response are 11 msec and 15 msec to the first peak of the evoked potential. This is consistent with a rapid high-bandwidth pathway, likely mediated by large-diameter fibers in the spinocerebellar tracts with transmission rates above 50 m/sec.

Although the maximum frequency generated by human voluntary motor output is < 20 Hz^21^, sensory stimuli may contain significantly higher frequencies. For example, a sudden displacement provides high-frequency step-function input, and the ability to determine relative force onset depends on the ability to respond to frequencies with periods much shorter than the reaction time. The faithful transmission of signals with high temporal frequency is therefore an important component of coordination, with particular salience for motor control and cerebellar computations involved with timing and precise high-speed movement.

The observed changes in amplitude may result from transmission bandwidth at any point in the pathway, sensitivity of thalamic circuits to specific frequencies, or a “paired-pulse” effect, with the penultimate pulse affecting the response to the final pulse. Thus, our data do not allow for a definitive conclusion regarding whether the observed effects were caused by the entire burst stimulation or by the final few pulses. This could be delineated in future experiments by varying the burst duration, or perhaps by comparison with stimulation within the thalamus or at intermediate points in the spinocerebellar-thalamic pathway.

High-frequency invasive electrical stimulation of the Voa/Vop, VIM, and VPL may effectively treat the symptoms of movement disorders such as essential tremor, and dystonia^6,22–25^, including secondary (acquired) dystonia in a subset of adolescents with cerebral palsy^4^. Previous studies have shown that stimulation of the PNS can elicit robust low-latency EPs in sensory thalamic areas such as VIM and VPL nuclei^19,20,26^. Stimulation of the PNS elicits attenuated low-latency EPs in motor projections such as the Voa/Vop nucleus^27^. However, no previous study has confirmed the ability to transmit high-frequency stimulation from the periphery to the thalamus. High-frequency transmission is important if we are to consider the use noninvasive techniques to emulate the high-frequency stimulation shown to be effective invasively.

We did not observe any clinical effects (beneficial or otherwise) in this study, but we note that the intermittent burst stimulation (needed in order to evaluate the pulse amplitude in the absence of artifacts) does not correspond to typical clinically effective stimulation patterns used in thalamus. Furthermore, the short duration of stimulation in this study is unlikely to have significant effects. In order to test for effects on dystonia, children would have had to attempt voluntary movement which would introduce significant motion artifacts. None of the children in this study had resting or postural tremor, and thus the effect on tremor could not be studied in this population. Future clinical trials based on the results here will be needed to determine if longer-term continuous peripheral stimulation can yield results similar to those seen with invasive stimulation.

The modulation of sensory pathways may also be effective in some cases of chronic pain^28^. Although it was not a primary goal of this study, the observation of EPs in VPL suggests that the use of peripheral electrical stimulation to modulate sensory transmission to thalamic nuclei could be effective in the management of pain syndromes.

An important caveat for interpretation of this study is that we examined the effects of peripheral stimulation only in adolescents undergoing deep brain stimulation for severe movement disorders, including dystonia. Peripheral sensory function and central sensory perception in these adolescents may not reflect the responses of healthy individuals or children or adults with other disorders. Furthermore, temporary implantation of intracranial electrodes could itself alter thalamic response to peripheral stimulation. Despite these caveats, we are encouraged that these results may generalize beyond our patients by noting that the latency and shape of the EPs, are consistent with previous reports on microelectrode recordings from adults^27^.

## Conclusion

In summary, our results demonstrate rapid and faithful transmission of high-frequency pulse sequences from peripheral nerve to the VIM nucleus of the thalamus. We observed changes in EP size as a function of pulse frequency, suggesting the existence of optimal stimulation frequencies. Our results identify the use of peripheral stimulation as a potential non-invasive alternative to high-frequency DBS for the activation of sensory thalamic nuclei. Further studies will be necessary to determine whether clinical efficacy can be achieved.

## Materials and methods

### Subjects

Ten pediatric patients undergoing deep brain stimulation (DBS) implantation for the treatment of dystonia were recruited to participate in the present study (Table 1). In each case, the diagnosis of dystonia was established by a pediatric movement disorder specialist (TDS) using standard criteria^29^.

**Table 1:** Demographic characteristics.

Subject	Diagnosis	Gender	Age	Leads
NMU1^a^	CP	F	15	VA; VIM; Voa/Vop
NMU2	CP	M	9	VA; VIM; Voa/Vop
NMU3	CP	M	21	VA; VIM; Voa/Vop
NMU4	CP	F	6	VIM; Voa/Vop
NMU5	CP	M	18	VIM; Voa/Vop
NMU6	CP	M	14	VPL; VIM; Voa/Vop
NMU7	CP	F	16	VA; VIM; Voa/Vop
NMU8	CP	M	20	VA; VIM; Voa/Vop
NMU9	CP	M	19	VPL; VIM; Voa/Vop
NMU10	CP	M	12	VA; VIM; Voa/Vop

*CP* cerebral palsy, *F* female, *M* male, *VPL* ventral posterolateral nuclei, *VA* ventral anterior, *VIM* ventralis intermediate, *Voa/Vop* ventral oralis anterior/posterior^30^.

^a^Subjects were identified from among the population of patients undergoing treatment in the neuromodulation monitoring unit (NMU) at Children’s Hospital of Los Angeles.

### Ethical approval

This study conformed to the ethical guidelines of the Declaration of Helsinki, and the protocol was approved by the University of Southern California Human Subjects Institutional Review Board (approval UP-13–00,521).

### Consent to participate

All patients signed informed consent for surgical procedures according to standard hospital policies (Children’s Hospital Los Angeles). Patients or parents of minor patients also sign research informed consent and assent for median nerve electrical stimulation and research use of electrophysiological data. Patients or parents of minor patients also sign Health Information Portability and Accountability Act (HIPAA) authorization for the research use of protected health information (PHI).

### Surgical procedure

Our standard clinical procedure for determining DBS targets includes the implantation of 6–10 temporary AD-TECH MM16C depth electrodes (AD-TECH MEDICAL INSTRUMENT CORPORATION, Oak Creek, WI, USA) at potential DBS targets (including basal ganglia and thalamic nuclei), as identified based on clinical criteria in each patient^3^. Typical thalamic targets for adolescents with dystonia include ventral oralis anterior/posterior (Voa/Vop), ventralis intermediate (VIM), ventral anterior (VA) and ventral posterolateral nuclei (VPL). The depth electrodes were placed using standard stereotactic procedure for the implantation of DBS electrodes, with the most distal stimulation contact placed at the target location. Electrode location was confirmed by co-registration of the preoperative T1-weighted and postoperative computed tomography (CT) scans, and further confirmed using the presence or absence of evoked potentials (EPs) induced during peripheral stimulation at low frequency^18^.

### VIM DBS electrode localization

Preoperative T1-weighted (anatomy) volumes were acquired from a MAGNETOM 3 T (SIEMENS Medical System, Erlangen, Germany) scanner for precise anatomical localization. The postoperative computerized tomography (CT) volumes were acquired for all patients from a GE (GENERAL ELECTRIC Healthcare, Milwaukee, WI, USA) scanner. Both the T1-weigthed image and the CT scan were co-registered and warped into MNI (Montreal Neurological Institute) space using Advanced Normalization Tools^31^. DBS electrode positions were corrected using the brain shift between the pre- and post-operative images^32^. DBS electrodes were pre-localized in native and MNI space using the PaCER algorithm^33^. A total of 20 VIM DBS trajectories on both cerebral sides for 10 dystonic patients were visualized through DSI Studio using the DISTAL atlas^14^.

### Electrophysiological recording

Recordings were performed during the first 24 to 48 h after clinical implantation of the temporary depth electrodes. Each electrode lead has a diameter of 1.2 mm and contains 6 low-impedance (1–2 kΩ) ring electrodes with 2 mm height and 5 mm spacing, as well as 10 high-impedance (70–90 kΩ) microelectrodes (50-µm diameter fine wire). The microelectrodes are arranged in groups of 2 or 3, spaced evenly around the circumference of the electrode shaft, between pairs of ring electrodes. The electrodes were connected to AD-TECH CABRIO connectors modified to include a custom unity-gain preamplifier for each microelectrode to reduce noise and motion artifacts. Macroelectrodes bypass the preamplifiers in order to allow for external electrical stimulation. All data reported here are from the high-impedance microelectrode recordings. Microelectrode signals were amplified, sampled at 24 kHz, and digitized by a TUCKER-DAVIS TECHNOLOGIES PZ5M analog-to-digital amplifier connected to an RZ2 digital signal processor. Data were streamed to an RS4 high-speed data storage unit, controlled by SYNAPSE recording software (SYSTEM3, TUCKER-DAVIS TECHNOLOGIES, Alachua, FL, USA).

### Stimulation protocol

Thalamic EPs were recorded during stimulation of the median nerve at the wrist, delivered through 1-cm adhesive disk electrodes with cathode proximal. Stimuli were generated in current-controlled mode by a STIMISOLA isolated stimulator (BIOPAC Systems Inc., Goleta, CA, USA) controlled by a Power1401-3A digital-to-analog converter (CAMBRIDGE ELECTRONIC DESIGN LIMITED, Cambridge, UK). Stimuli consisted of 1-ms biphasic pulses delivered at 5 Hz (single-pulse stimulation) or as 100-ms pulse trains at 50 Hz, 100 Hz, 140 Hz, and 170 Hz, repeated every 200 ms. With this protocol, each 100-ms burst was followed by 100 ms without stimulation. The amplitude of the stimulus was adjusted to the minimum current that produces a palpable twitch in thenar muscles, while remaining below the threshold of discomfort. This resulted in average stimulation current of 6.46 mA $\pm$ 2.1SD. Recordings were collected for a period of 4 min. Approximately 1200 pulse trains were delivered for each stimulation frequency.

No adverse events were noted. Movement was not tested during the stimulations, and thus no beneficial effects were noted. Two of the subjects were unable to tolerate 170 Hz stimulation due to discomfort.

### Data analysis

All microelectrode signals were processed offline in Matlab (Matlab, R2019b). A digital high-pass filter (5 Hz, 4th order, Butterworth) was applied to all microchannels. Stimulation artifacts were identified by threshold detection of transients that exceed 4 standard deviations from the background, and evoked responses were time aligned to the artifact peaks. Stimulus pulse trains were segmented and averaged for each stimulation frequency. Baseline drift following each evoked response was removed by estimating and subtracting the best-fit 5^th^-order polynomial. Minimum and maximum normalization following the last stimulus artifact of each burst were applied. For each frequency, the average time of occurrence of the maximum and minimum values between 10 and 20 ms following the last stimulus artifact of each burst was determined, and the EP amplitude was determined as the average difference in amplitudes at these times for each frequency:

1 $${V}_{EP}(f)=V\left({t}_{max}(f)\right)-V\left({t}_{min}(f)\right)$$

where ${V}_{EP}(f)$ denotes EP amplitude, ${t}_{max}(f)$ denotes the average time of the EP peak for the stimulation frequency, and ${t}_{min}$(f) denotes the average time of the EP minimum.
